# Combating castration-resistant prostate cancer by co-targeting the epigenetic regulators EZH2 and HDAC

**DOI:** 10.1371/journal.pbio.3002038

**Published:** 2023-04-27

**Authors:** Amy E. Schade, Ryan Kuzmickas, Carrie L. Rodriguez, Kaia Mattioli, Miriam Enos, Alycia Gardner, Karen Cichowski

**Affiliations:** 1 Department of Medicine, Division of Genetics, Brigham and Women’s Hospital, Boston, Massachusetts, United States of America; 2 Harvard Medical School, Boston, Massachusetts, United States of America; 3 Ludwig Center at Harvard, Harvard Medical School, Boston, Massachusetts, United States of America; Consejo Nacional de Investigaciones Científicas y Técnicas: Consejo Nacional de Investigaciones Cientificas y Tecnicas, ARGENTINA

## Abstract

While screening and early detection have reduced mortality from prostate cancer, castration-resistant disease (CRPC) is still incurable. Here, we report that combined EZH2/HDAC inhibitors potently kill CRPCs and cause dramatic tumor regression in aggressive human and mouse CRPC models. Notably, EZH2 and HDAC both transmit transcriptional repressive signals: regulating histone H3 methylation and histone deacetylation, respectively. Accordingly, we show that suppression of both EZH2 and HDAC are required to derepress/induce a subset of EZH2 targets, by promoting the sequential demethylation and acetylation of histone H3. Moreover, we find that the induction of one of these targets, *ATF3*, which is a broad stress response gene, is critical for the therapeutic response. Importantly, in human tumors, low *ATF3* levels are associated with decreased survival. Moreover, *EZH2*- and *ATF3-*mediated transcriptional programs inversely correlate and are most highly/lowly expressed in advanced disease. Together, these studies identify a promising therapeutic strategy for CRPC and suggest that these two major epigenetic regulators buffer prostate cancers from a lethal response to cellular stresses, thereby conferring a tractable therapeutic vulnerability.

## Abstract 2

Epigenetic, transcriptional, and functional studies show that two major epigenetic regulators (EZH2 and HDAC) buffer advanced prostate cancer from lethal stress responses, revealing a promising combination therapy for castration-resistant disease.

## Introduction

The development of prostate cancer is dependent on androgen receptor (AR) signaling [1]. However, while androgen deprivation therapy is used to treat advanced disease, tumors that are refractory to anti-androgen therapies or are completely androgen independent can emerge [2,3]. Consequently, prostate cancer remains the third-leading cause of cancer death in men [2]. Therefore, it may be critical to develop therapeutic strategies for castration-resistant prostate cancer (CRPC) that extend beyond targeting AR pathways.

Relatively little is known about the signals that drive and maintain advanced CRPC. However, one gene implicated in various aspects of prostate cancer progression is *EZH2* (*enhancer of zeste homologue 2*). *EZH2* encodes the histone methyltransferase component of the Polycomb Repressive Complex 2 (PRC2), which regulates epigenetic gene silencing [4]. It is overexpressed and/or amplified in prostate cancer, and expression levels are highest in advanced, metastatic tumors [5]. Moreover, EZH2 plays a direct causal role in driving prostate tumor progression and metastasis in animal models [6]. EZH2 has also been implicated in maintaining a neuroendocrine-like state in a subset of CRPCs, characterized, in part, by the loss of AR expression and an acquisition of AR independence [7]. These distinct roles for EZH2 in promoting prostate cancer progression, metastasis, and AR independence raise the possibility that it could be a potential therapeutic target in advanced disease.

Agents that inhibit EZH2 are being clinically evaluated in a variety of tumor types [8], and one EZH2 inhibitor has been approved for the treatment of epithelioid sarcomas [9,10]. However, in animal and cell culture models of prostate cancer, EZH2 inhibitors modestly reduce proliferation, but exhibit little efficacy as single agents [7,11]. In neuroendocrine-like CRPC models, EZH2 inhibition reverses the neuroendocrine phenotype, restores AR expression, and partially resensitizes cells to androgen deprivation therapy [7]. Nevertheless, while combined AR and EZH2 inhibitors exert a more potent cytostatic response in these models, tumors still do not regress. Therefore, we set out to identify an EZH2 inhibitor-based drug combination that was capable of killing CRPC and inducing frank tumor regression.

The PRC2 complex confers a repressive transcriptional signal by methylating histone H3 at lysine 27 (H3K27me3). Accordingly, EZH2 inhibitors promote a loss of this repressive mark [12]. However, the loss of this repressive signal may not be sufficient to restore transcription at some PRC2-regulated sites, if H3K27 acetylation (H3K27ac), which is associated with transcriptional activation, is suppressed. Interestingly, histone deacetylases (HDACs) are also overexpressed in prostate cancer and physically interact with PRC2 [13–15]. Therefore, HDACs often work in concert with the PRC2, by removing histone acetylation marks so that methylation may occur. As such, we hypothesized that the combined suppression of both EZH2 and HDAC might be required to derepress an important subset of PRC2 target genes in CRPC, by promoting the demethylation and the subsequent acetylation of H3K27 at some promoters. Here, we show that this is indeed the case. More importantly, we have discovered that EZH2 and HDAC inhibitors kill prostate cancers, in part, by activating a broad stress response gene, which is normally repressed in advanced tumors.

## Results

### Combined EZH2 and HDAC inhibitors kill CRPC

**Fig 1:**
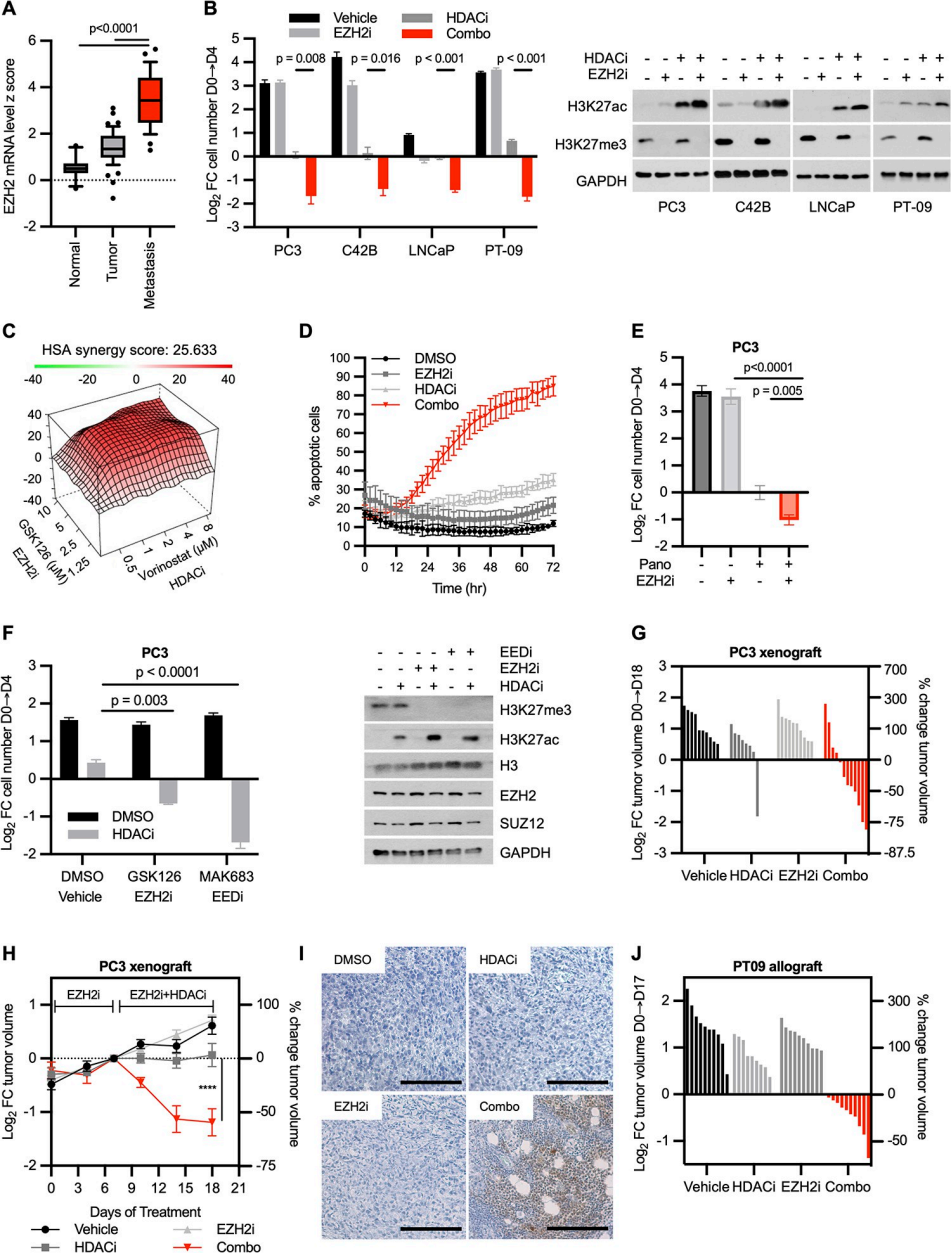
Combined EZH2 and HDAC inhibitors kill CRPC cells and trigger regression of CRPCs in vivo. **(A)** Box plot displaying z score values of EZH2 mRNA expression in normal prostate, primary prostate adenocarcinoma, and metastatic prostate cancer tumors. Data obtained from Grasso and colleagues [45] (S1 Data). (**B)** Bar graph illustrating the cytotoxic effects of EZH2 and HDAC inhibitors. Three human prostate cell lines (PC3, C42B, and LNCaP) and one mouse cell line (PT-09) were treated with DMSO, 5 μM GSK126 (EZH2i), 2 μM vorinostat (HDACi), or both agents combined as described. Manual cell counting was performed on day 0 and on day 4 after combined treatment. Graphs (left) show log_2_ FC in cell number on day 4 compared to day 0. Immunoblots (right) confirm target inhibition (S1 Data). (**C)** Synergy plot using Gaddum’s noninteraction model (HSA) for PC3 cells treated with EZH2i (GSK126) and/or HDACi (vorinostat). Plot is representative of 3 replicates. (**D**) Graph depicting percentage of caspase 3/7+ (apoptotic) cells over time using Incucyte live cell imaging (S1 Data). (**E)** Bar graph illustrating the cytotoxic effects of panobinostat and GSK126. PC3 cells were treated with DMSO, 5 μM GSK126 (EZH2i), 20 nM Panobinostat (Pano), or a combination of the 2 inhibitors. Log_2_ FC was calculated as in Fig 1B (left) (S1 Data). (**F**) Bar graph illustrating the cytotoxic effects of combined EED/HDAC inhibitors. PC3 cells were treated with DMSO, 2 μM vorinostat (HDACi), 5 μM GSK126 (EZH2i), 5 μM MAK683 (EEDi), or combined agents as denoted. Log_2_ FC was calculated as in Fig 1B (left) (S1 Data). Immunoblots (right) show target inhibition in each experimental group at 24 hours posttreatment. (**G**) Waterfall plot depicting log_2_ FC in tumor volume of PC3 xenografts after 18 days of treatment with EZH2i (GSK126) and/or HDACi (vorinostat). (*n =* 8–12 per arm) (S1 Data). (**H**) Tumor growth curve of PC3 xenograft from (G) over 18 days of treatment. The combination treatment arm contains only tumors that responded to the treatment so that the kinetics of regression could be visualized (*n* ≥ 8 tumors per arm) (S1 Data). (**I**) Immunohistochemistry of CC3 in PC3 xenografts after 11 days of treatment (7 days pretreatment ±EZH2i plus 4 days ±HDACi). (**J**) Waterfall plot depicting log_2_ FC in tumor volume of mouse PT-09 allografts after 18 days of treatment with EZH2i (GSK126) and/or HDACi (vorinostat). (*n =* 10 per arm) (S1 Data). For all subfigures, data are mean ± SD of biological independent samples, except for in vivo data, which are reported as mean ± SE. *P* values measured by unpaired one-tailed heteroscedastic Student *t* test. CC3, cleaved caspase 3; CRPC, castration-resistant prostate cancer; EZH2, enhancer of zeste homologue 2; FC, fold change; HDAC, histone deacetylase.

*EZH2* is overexpressed in prostate cancer and protein levels appear to progressively increase in advanced tumors [5]. Analysis of transcriptional profiles from a panel of prostate cancers and normal prostate tissue confirms this pattern of expression and reveals that 92% of metastatic samples express *EZH2* at levels ≥3 SD higher than those observed in normal prostate tissue (Fig 1A and S1 Data; [16]). Nevertheless, while EZH2 plays a causal role in driving prostate cancer progression and metastasis [6], EZH2 suppression alone is not sufficient to kill prostate cancer cells in vitro or in vivo [7,11]. Given the functional interaction between PRC2 and HDACs, and the concept that the transcription of specific genes might require both a loss of H3K27me3 and concomitant gain of H3K27ac, we reasoned that the suppression of both enzymes might be required for the up-regulation of a subset of important PRC2 targets. If so, these agents might exert more potent therapeutic effects when combined.

To investigate the biochemical and biological consequences of EZH2 and HDAC inhibitors in prostate cancer, we used a panel of cell lines including PC3 (AR-), C42B (AR independent), LnCAP (AR dependent), and PT-09 cells (mouse, AR independent). Cells were pretreated with the EZH2 inhibitor GSK126 for 5 days to permit the accumulation of demethylated H3K27, as previously described [17], followed by the addition of the HDAC inhibitor, vorinostat. GSK126 alone exerted either no or modest effects on cell proliferation, whereas vorinostat conferred more potent cytostatic responses (Fig 1B and S1 Data). Strikingly, combined suppression of EZH2 and HDAC triggered a dramatic loss of cells in all 4 models (Fig 1B and S1 Data). As expected, inhibition of EZH2 effectively suppressed H3K27me3; however, we noted that combined EZH2 and HDAC inhibitors cooperatively increased H3K27ac levels (Fig 1B, right panel). Importantly, EZH2 and HDAC inhibitors potently synergized with HSA scores well above 10 (Fig 1C). EZH2 inhibition also substantially reduced the IC_50_ value of the HDACi, vorinostat (S1A Fig and S2 Data). Finally, live cell imaging using a caspase 3/7 reporter revealed that these agents triggered high levels of apoptosis when combined, killing 80% to 90% of cells within just 3 days (Fig 1D and S1 Data).

The cytotoxic effects of the combination could be recapitulated by substituting vorinostat with another Class I HDAC inhibitor, panobinostat (Fig 1E and S1 Data), or by replacing GSK126 with MAK683, an agent that suppresses a different obligate PRC2 component, EED (Fig 1F and S1 Data). Importantly, combined EZH2 and HDAC inhibitors were not generally cytotoxic as they did not kill immortalized prostate epithelial cells or other unrelated cell lines (S1B and S1C Fig and S2 Data). It is notable that these agents were effective both in castration-resistant models that either expressed (C4-2B) or lacked AR (PC3 cells), suggesting that the response was independent of any potential effects on AR signaling.

### EZH2 and HDAC inhibitors trigger the regression of CRPC in vivo

We next evaluated the efficacy of these agents in vivo. To eliminate any potential effects of AR signaling, we used PC3 xenografts, which do not express the AR. Animals with established tumors (100 to 200 mm^3^) were either pretreated with the EZH2 inhibitor or vehicle to permit loss of H3K27me3. After 7 days of the pretreatment phase, mice were further segregated into 4 treatment arms (vehicle, HDACi, EZH2i, or HDAC/EZH2i) and were exposed to drugs for a total of 18 days. Consistent with in vitro observations, EZH2 and HDAC inhibitors exerted minimal effects on their own, but when combined triggered potent tumor regression (Fig 1G and S1 Data). Analysis of H3K27me3 and H3K27ac in tumor tissue confirmed that both agents effectively suppressed their targets in vivo (S1D Fig). Growth curves of responsive tumors demonstrate that EZH2 inhibition had no effect on tumor growth alone during the pretreatment phase, but that tumors immediately began to regress once HDACi was included in the regimen, and continued to shrink over time (Fig 1H and S1 Data). It should be noted that PC3 xenografts are an extremely aggressive tumor model, and few, if any, agents have been shown to cause this type of response. Cell death was readily observed within 4 days in tumors treated with both agents, as demonstrated by extensive cleaved caspase 3 (CC3) staining, which was not detected in tumors treated with vehicle or each single agent alone (Fig 1I). Notably, EZH2 and HDAC inhibitors also triggered tumor regression in an immunocompetent mouse model of AR-independent prostate cancer, PT-09, indicating that this combination remains effective in the context of an intact immune system (Figs 1J and S1E and S1 and S2 Data) [18].

### EZH2 and HDAC inhibitors cooperatively derepress a subset of PRC2 targets in CRPC

**Fig 2:**
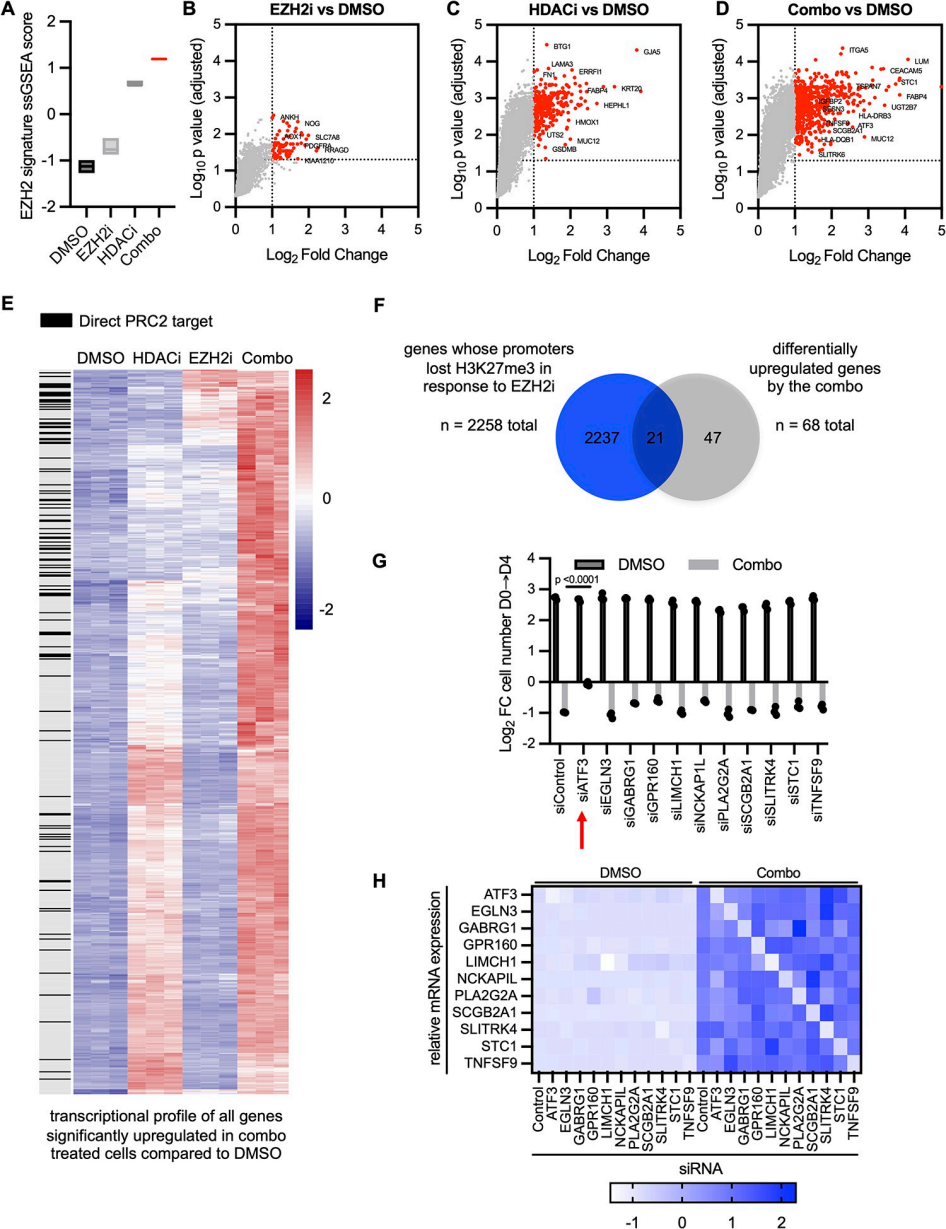
EZH2 and HDAC inhibitors cooperatively derepress a subset of PRC2 targets in CRPC. **(A)** Single sample GSEA of PC3 cells treated with DMSO, EZH2i, HDACi, or the combination. Box plot shows min to max of z scores with line at median for each treatment (S2 Data). (**B-D)** Volcano plots depicting up-regulated genes in EZH2i vs. DMSO (**B**), HDACi vs. DMSO (**C**), or Combo vs. DMSO (**D**) samples (S2 Data). Each dot is one gene. Red dots are significantly differentially expressed genes (log_2_ fold change >1, adjusted *p*-value <0.05). (**E)** Heatmap showing the transcriptional profile of all genes significantly up-regulated in combo-treated cells vs. DMSO (log_2_ fold change >1, adjusted *p*-value <0.05). Each gene is annotated for whether it is a direct PRC2 target (gene whose promoter lost H3K27me3 in response to EZH2i). (**F)** Venn diagram of potential PRC2 targets (e.g., genes whose promoters lost H3K27me3 in response to EZH2i) and genes differentially induced by the combination (significantly different genes in all 3 comparisons: Combo vs. DMSO, Combo vs. HDACi, Combo vs. EZH2i). (**G**) Bar graph depicting functional screen of top candidate genes identified in **F**. PC3 cells were transfected with the indicated siRNAs and then treated with DMSO or Combo. Relative cell counts are shown in relation to day 0 (S2 Data). Each value is reported as mean ± SD of 3 biologically independent samples. *P* value measured by unpaired one-tailed heteroscedastic Student *t* test. (**H)** Heatmap confirming knockdown of targets in **G**. RT-qPCR analysis of indicated mRNAs was performed in response to the combination in PC3 cells transfected with the indicated siRNAs. EZH2, enhancer of zeste homologue 2; GSEA, gene set enrichment analysis; HDAC, histone deacetylase; PRC2, Polycomb Repressive Complex 2; RT-qPCR, quantitative reverse transcription PCR.

Our overarching hypothesis was that EZH2 and HDAC inhibitors might be transducing their effects by cooperatively derepressing and up-regulating critical PRC2 targets. To investigate this possibility, ssGSEA was performed on mRNA expression data obtained from PC3 cells exposed to vehicle, EZH2i, HDACi, or the combination. Notably, the drug combination significantly up-regulated a previously described PRC2 signature to a greater extent than either single agent alone, consistent with the hypothesis that these agents were cooperatively derepressing a subset of PRC2 targets (Fig 2A and S3 Data).

To characterize the broad transcriptional response to combined EZH2 and HDAC inhibitors in CRPC cells, and to parse out the effects on direct EZH2 targets, mRNA expression data were analyzed in conjunction with H3K27me3 chromatin immunoprecipitation-sequencing (ChIP-seq). First, we identified all transcripts that were significantly up-regulated by the single agents or the combination (Fig 2B–2D and S3 Data). Genes up-regulated by either EZH2 or HDAC inhibition alone are depicted by the red dots in Fig 2B and 2C, respectively; however, these agents triggered an even greater transcriptional response when combined (Fig 2D and S3 Data). A heat map of all genes that were significantly up-regulated in combination-treated cells illustrates the cooperative nature of these effects, as the majority required both agents for maximal expression, albeit to different extents (Fig 2E). Integration of H3K27me3 ChIP-seq data further demonstrated that a subset of these genes were direct PRC2 targets, denoted by black boxes (Fig 2E).

Based on these findings, we sought to determine whether any direct PRC2 targets that were uniquely derepressed in the presence of both EZH2 and HDAC inhibitors might contribute to the therapeutic response. To generate a list of candidates, we identified 2,258 potential PRC2 target genes, defined as those in which H3K27me3 was lost at promoter sequences in response to EZH2 inhibition (Figs 2F and S2A and S1 Table). We then compared this list to the 68 genes that were differentially and uniquely up-regulated by more than 2-fold in the presence of both EZH2 and HDAC inhibitors, versus single agents. Twenty-one overlapping candidates were identified using this strategy, as depicted by the Venn diagram in Fig 2F. A list and heatmap of all 68 genes, as well as their relative expression in response to single and combined agents, is shown (S2B Fig), with the 21 direct PRC2 targets listed in blue. These data further highlight the cooperative effects that EZH2 and HDAC inhibitors have on the expression of specific genes in CRPC cells, including a subset of direct PRC2 targets.

To determine whether any of these direct PRC2 targets were critical regulators of the therapeutic response, we performed a screen using pooled siRNAs recognizing each gene (Fig 2H). We prioritized genes with either unknown or potential tumor suppressive activity and deprioritized those that would not be expected to exert cell autonomous or tumor suppressive effects (e.g., HLA genes; S2C Fig). siRNAs were independently introduced into cells, which were then exposed to combined EZH2 and HDAC inhibitors. *ATF3* stood out among these candidates, as its loss potently suppressed the the cytotoxic response, thus warranting further study (Fig 2G and S3 Data). Knockdown efficiency of all siRNAs in the screen were confirmed by quantitative reverse transcription PCR (RT-qPCR) (Fig 2H).

### EZH2 and HDAC inhibitors kill CRPC by up-regulating the common stress responsive transcription factor, ATF3

**Fig 3:**
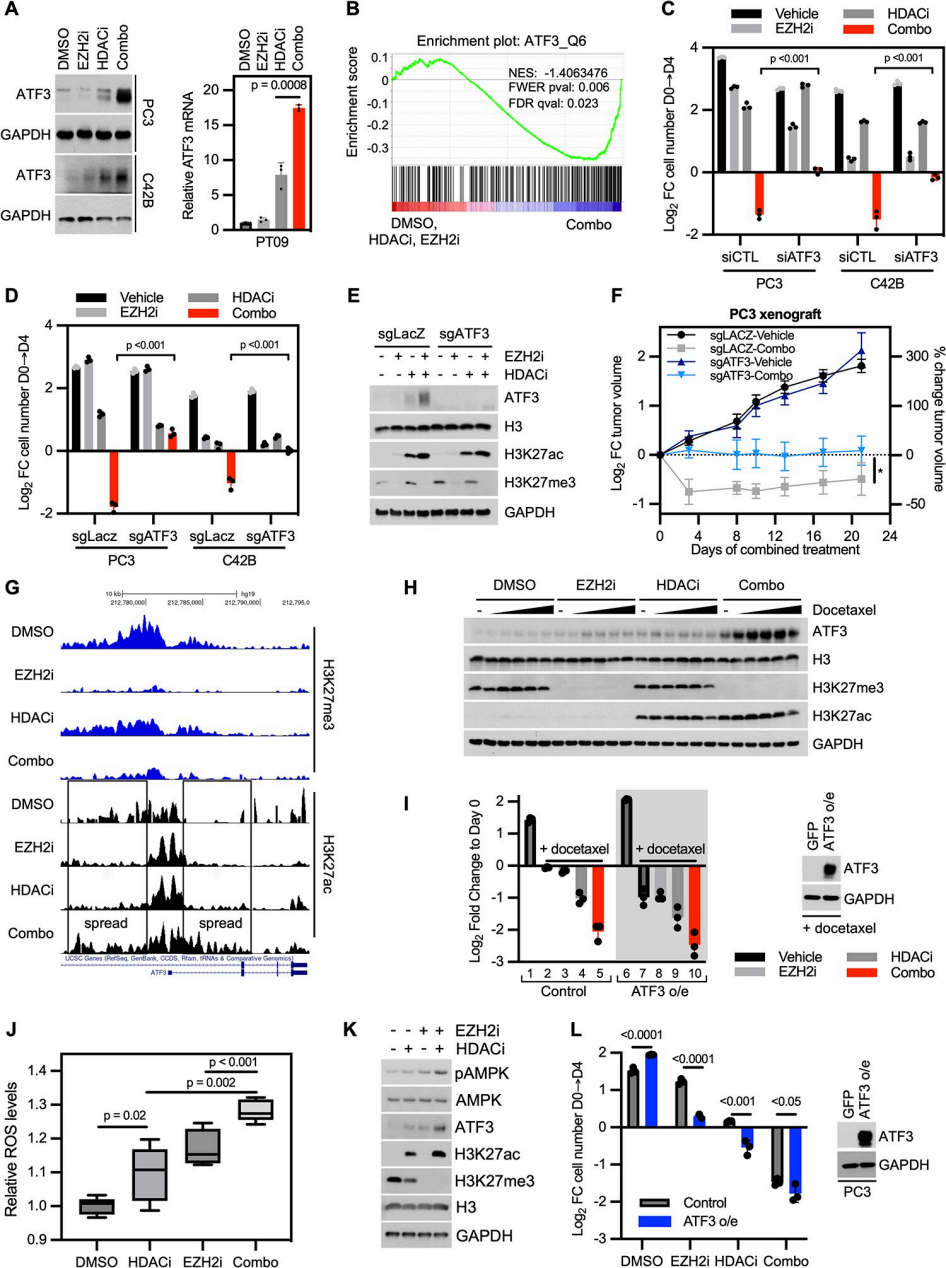
EZH2 and HDAC inhibitors kill CRPCs by up-regulating the common stress responsive transcription factor, ATF3. (**A)** Expression of ATF3 in reponse to EZH2 and HDAC inhibitors. Prostate cancer cell lines treated with EZH2i and/or HDACi. Immunoblot of PC3 and C42B human cell lines (left) or RT-qPCR of PT-09 mouse cell line (right) with values normalized to GAPDH and fold change calculated in reference to DMSO (S3 Data). (**B)** GSEA of ATF3 transcriptional gene set (ATF3_Q6) comparing combo-treated PC3 cells to DMSO-, EZH2i-, or HDACi-treated cells. (**C)** Graph depicting relative change in cell number of indicated cell lines transfected with siRNAs against a control sequence or ATF3 and then treated with vehicle, EZH2i, and/or HDACi (S3 Data). (**D)** Graph depicting relative change in cell number of indicated cell lines stably transduced with sgRNAs against a control sequence or ATF3 and then treated with vehicle, EZH2i, and/or HDACi (S3 Data). (**E)** Western blot of lysates from cells from (**D**) probed with the indicated antibodies. (**F)** Relative tumor growth curve of PC3 xenografts generated from cells described in (e) implanted subcutaneously in the flank of male castrated nude mice (*n =* 7–10 per arm) (S3 Data). *P* value is 0.015 and measured by two-way ANOVA test. (**G)** Genome browser view of the ATF3 transcriptional start site. Tracks for H3K27me3 are shown in blue and H3K27ac are shown in black. (**H)** ATF3 immunoblots from PC3 cells treated with DMSO, EZH2i, HDACi, or the combination. Protein lysates were harvested after cells were additionally treated with increasing doses of docetaxel (0, 1, 2, 4, 8, 10 nM) for 24 hours. (**I**) Bar graph of PC3 cells pretreated with EZH2i and then treated with HDACi and/or docetaxel (10 nM) or DMSO controls. Numbers are included for ease of reference. Note that cells represented by bars 2–5 and 7–10 were treated with docetaxel. Relative cell count after treatment was measured after 4 days of combination treatment (left) (S3 Data). Left (white) side of graph: Cells also expressed a GFP control vector. Right (gray) side of graph: Cells expressed ectopic ATF3. Confirmation of ATF3 overexpression is shown in DMSO-treated cells (right). (**J)** Box plot depicting relative levels of ROS to DMSO after 24 hours of treatment with EZH2i and/or HDACi in PC3 cells (S3 Data). Box plot reports the mean of 5 biologically independent samples with whiskers reporting minimum and maximum. *P* values measured by unpaired one-tailed homoscedastic Student *t* test. (**K)** Immunoblot measuring phosphorylated AMPK, a sensor of metabolic stress. Protein lysates collected from PC3 cells after 8 hours of combination treatment. (**L)** Bar graph depicting drug effects of PC3 cells stably transduced with ATF3 as in (**I**), but in the absence of docetaxel. Relative cell counts after treatment with EZH2i and/or HDACi was measured after 4 days of combination treatment (left) (S3 Data). Confirmation of ATF3 overexpression is shown in DMSO-treated cells (right). For all subfigures, data are mean ± SD of biological independent samples, except for in vivo data, which are reported as mean ± SE. Unless otherwise noted, *P* values measured by unpaired one-tailed heteroscedastic Student *t* test. EZH2, enhancer of zeste homologue 2; GSEA, gene set enrichment analysis; HDAC, histone deacetylase; ROS, reactive oxygen species; RT-qPCR, quantitative reverse transcription PCR.

ATF3 is common stress response transcription factor [19]. Its expression is rapidly induced by multiple cellular stresses including oxidative stress, metabolic stress, ER stress, DNA damage, and a variety of environment signals [19]. Moreover, sustained ATF3 up-regulation has been shown to be a critical mediator of cell death in response to many of these insults and some chemotherapies [20–24]. Therefore, we examined the expression pattern of ATF3 in response to EZH2 and HDAC inhibitors. Notably, the ATF3 protein was minimally expressed in vehicle and EZH2 inhibitor treated cells, and its expression was slighly increased in HDAC-treated cells; however, ATF3 was dramatically up-regulated in response to the drug combination in PC3 and C4-2B cells (Fig 3A and S4 Data). A similar expression pattern was observed at the mRNA level in these cell lines and in PT-09 cells, illustrating the potent up-regulation of *ATF3* in mouse CRPC cells, as the protein was not effectively recognized by the anti-human ATF3 antibody (Figs 3A and S3A, S4 and S5 Data files). Gene set enrichment analysis (GSEA) of data from the 4 treatment conditions further revealed that the ATF3 transcriptional signature was significantly enriched in cells treated with combined EZH2/HDAC inhibitors (Figs 3B and S3B), suggesting that this pathway was being activated by these agents.

To further validate ATF3 in the response to EZH2 and HDAC inhibitors, *ATF3* siRNAs and CRISPR sgRNAs were introduced into both PC3 and C4-2B cells. In all cases, suppression of *ATF3* prevented cell death induced by the combination (Figs 3C, 3D and S3C and S4 Data). Moreover, ablation of *ATF3* prevented tumor regression in response to EZH2 and HDAC inhibitors in vivo (light blue line, Fig 3E and 3F and S4 Data). Together, these studies demonstrate that ATF3 is potently induced by EZH2 and HDAC inhibitors and is a critical mediator of the therapeutic response in vitro and in vivo.

### EZH2 and HDAC inhibition are required for maximal ATF3 expression

Next, we assessed the effects of EZH2 and HDAC inhibitors on H3K27me3 and H3K27ac binding at the *ATF3* promoter. ChIP-seq analysis revealed H3K27me3 binding at the *ATF3* promoter in vehicle-treated cells, which was lost in response to EZH2 and EZH2/HDAC inhibitors (Fig 3G), demonstrating that *ATF3* is a direct PRC2 target. Interestingly, however, while HDACi and EZH2i each slightly increased the major H3K27ac peaks at the ATF3 promoter, when combined, these agents triggered a robust spreading of the H3K27ac signal, both 5′ and even more so into the gene body, which largely overlapped with the boundaries of H3K27me3 in untreated cells (Fig 3G). Indeed, HDAC inhibitors are known to induce spreading of H3K27ac signal into the gene body of responsive genes [25], which is associated with their increased transcription [26]. Thus, collectively, our findings demonstrate that both EZH2 and HDAC inhibition are necessary for maximal ATF3 expression in CRPC cells and suggest that this is due to the sequential and combined effects on H3K27me3 (loss) and H3K27ac (spreading), respectively.

To provide additional evidence that the direct effects of EZH2 and HDAC inhibitors on chromatin were important for ATF3 induction and cell death, we assessed the effects of docetaxel, in the presence and absence of EZH2 and HDAC inhibitors. ATF3 is normally induced by cell stress, and its expression can be up-regulated by chemotherapy in other settings, because it triggers stresses such as reactive oxygen species (ROS), DNA damage, etc. [20–24]. However, we reasoned that if EZH2 and HDAC enzymes were insulating the ATF3 locus in CRPCs, its expression might be restrained.

Cells were treated with EZH2 and/or HDAC inhibitors, along with increasing concentrations of docetaxel (Fig 3H). As shown throughout, EZH2i alone did not induce ATF3, whereas EZH2i plus docetaxel resulted in a very subtle increase in ATF3 expression (Fig 3H). HDACi and docetaxel also stimulated a slight increase in ATF3 levels. However, these relatively low levels of ATF3, despite the presence of increasing concentrations of docetaxel, suggested that chromatin modifications might be restricting its maximal induction (Fig 3H). Consistent with this notion, while combined EZH2 and HDAC inhibitors were sufficient to induce ATF3 expression, when both agents were present, the further addition of docetaxel triggered a massive induction of ATF3 (Fig 3H). These findings suggest that the chromatin changes triggered by EZH2 and HDAC permit ATF3 expression, which can be further enhanced by cell stress signals, such as those triggered by chemotherapy. Notably, both EZH2 and HDAC inhibitors were required for this response.

The cooperativity between combined EZH2i/HDACi and chemotherapy was also evident in cell counting experiements. Docetaxel alone exerted a potent cytostic effect (Fig 3I and S4 Data, bar 2 versus bar 1), which was not further affected by the addition of EZH2i (Fig 3I and S4 Data, bar 3). Surprisingly, docetaxel and HDACi did cooperatively induce a loss of cells (Fig 3I and S4 Data, bar 4), which may reflect the slightly higher levels of ATF3 typically induced by HDACi (Fig 3A and 3E and S4 Data). Nevertheless, together, docetaxel, EZH2i, and HDACi triggered the greatest cytotoxic response (Fig 3I and S4 Data, bar 5).

Conversely, we reasoned that if EZH2 and HDACs normally restrain ATF3 expression, then ectopic ATF3 overexpression should minimize the need for its derepression. Exogenous ATF3 had no effect on its own, as expected, because cell death requires a stress signal (Fig 3I, bar 6, and S4 Data). Interestingly, however, ATF3 expression enhanced the effects of chemotherapy, resulting in cell death and a loss of cells (Fig 3I, bar 7, and S4 Data). Consistent with single agent responses, EZH2i exerted no further effect (Fig 3I, bar 8, and S4 Data), while HDACi slightly potentiated cell death (Fig 3I, bar 9, and S4 Data). Nevertheless, in the presence of ectopic ATF3, combined EZH2/HDAC/docetaxel exerted the most potent cytotoxic response (Fig 3I, bar 10, and S4 Data). These results demonstrate that ATF3 can readily kill CRPC cells in the presence of exogenous cell stress. However, the observation that EZH2 and HDAC inhibitors further enhanced the effects of chemotherapy (+ATF3), suggested that these agents might also be contributing additional stress signals (Fig 3I, bar 10, and S4 Data).

### EZH2 and HDAC inhibitors also enhance cell stress in CRPCs

Combined EZH2 and HDAC inhibitors clearly induce ATF3 expression in CRPC cells (Fig 3A, 3E and 3H and S4 Data). However, ATF3-mediated death also requires cellular stress. This fact, together with findings in Fig 3I, led us to investigate whether EZH2 and HDAC inhibitors might also be enhancing cell stress in these cells. Of note, HDAC and EZH2 inhibitors have individually been reported to trigger cell stress in other settings, such as oxidative stress, DNA damage, proteotoxic stress, and metabolic stress, in part by suppressing protective pathways (antioxidant pathways, DNA repair, etc.) [27–31].

Importantly, we found that EZH2 and HDAC inhibitors individually and cooperatively triggered ROS production, a driver of oxidative stress (Fig 3J and S4 Data). These agents also similarly activated AMPK, a marker of metabolic stress within just 8 hours (Fig 3K), which was accompanied by a dramatic reduction in ATP levels at this early time point, well prior to cell death (S3D Fig and S5 Data). These findings suggest that, in addition to directly promoting changes in chromatin at the ATF3 locus that enhance transcription, EZH2 and HDAC inhibitors also induce multiple cellular stresses, which may also contribute to the therapeutic response. Consistent with this notion, even in the absence of chemotherapy, ectopic expression of ATF3 enhanced the individual biological effects of EZH2 and HDAC inhibitors in cell counting assays (Fig 3L and S4 Data). ATF3 overexpression also potentiated the effects of combined EZH2/HDAC inhibitors, although cell death was already near maximal with the combination of EZH2 and HDAC inhibitors alone. Taken altogether, these studies suggest that EZH2 and HDAC inhibitors induce ATF3 expression and kill CRPCs by (1) derepressing the ATF3 locus and (2) cooperatively enhancing cell stress signals.

### Low ATF3 expression is associated with decreased survival and EZH2 and ATF3 signatures inversely correlate in human prostate cancers

Our in vitro studies suggest that ATF3 is normally suppressed by the PRC2 complex in CRPC and that this suppression is reinforced by HDACs. In the context of prostate tumor development and progression, we speculate that the epigenetic suppression of *ATF3* by EZH2 by might be an important protective mechanism that promotes survival as tumors develop/progress and are subject to increasing cellular and environmental insults. Consistent with this notion, genetic ablation of *ATF3* in genetically engineered mouse prostate cancer models has been shown to increase the rate of prostate tumor development and progression and decreases apoptosis in tumors [32,33]. Therefore, we investigate the relationship between ATF3 and PRC2 activity in human tumors.

**Fig 4:**
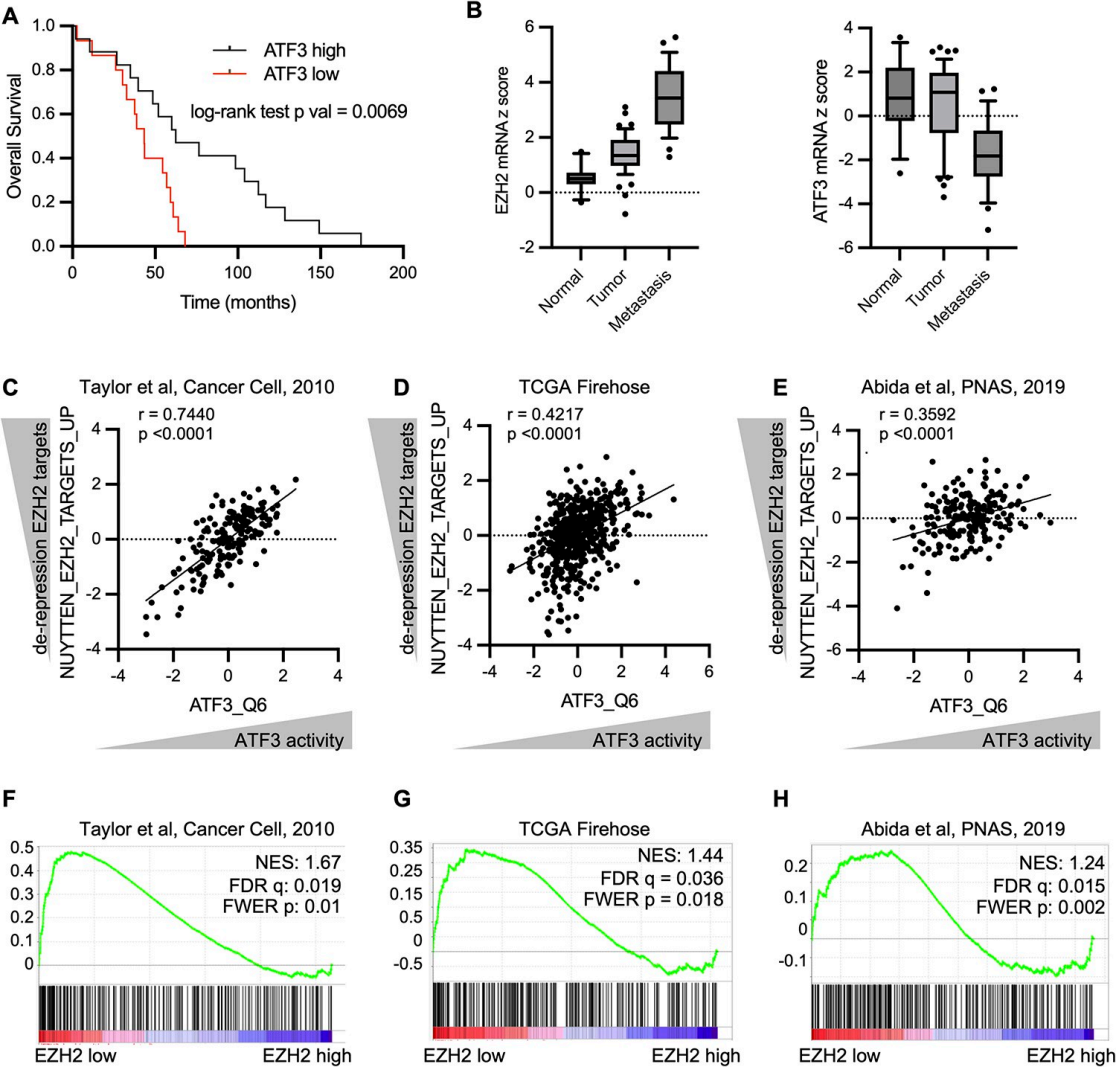
Low ATF3 expression is associated with decreased survival and EZH2 and ATF3 signatures inversely correlate in human prostate cancers. **(A**) Kaplan–Meier survival curve depicting survival of patients with high *ATF3* transcript expression (top 10% of all samples) vs. patients with low ATF3 transcript expression (bottom 10% of all samples) (S4 Data). (**B**) Box plot depicting expression of EZH2 mRNA (left) or ATF3 mRNA (right) in normal prostate, primary prostate adenocarcinoma, or metastatic prostate cancer (S4 Data). Box plot of EZH2 mRNA is same as in Fig 1A for comparison purposes. Data obtained from Grasso and colleagues [45]. (**C-E**) Correlation plots of ATF3 transcriptional sigantures (ssGSEA for ATF3_Q6) compared to EZH2 transcriptional signatures (ssGSEA for NUYTTEN_EZH2_TARGETS_UP) in 3 studies. An increase in the NUYTTEN signature indicates increased derepression of EZH2 targets. An enrichment of ATF3 signatures is depicted as increased ATF3 activity. R value is the Pearson correlation coefficient (S4 Data). (**F-H**) GSEA of ATF3 transcriptional program (gene set: ATF3_Q6) in EZH2 high and EZH2 low tumors in 3 datasets. EZH2, enhancer of zeste homologue 2; FDR, false discovery rate; FWER, family-wise error rate; GSEA, gene set enrichment analysis; NES, normalized enrichment score.

Analysis of transcriptional profiles from primary human prostate cancers revealed that high levels of *ATF3* levels are associated with improved overall survival (Fig 4A and S6 Data). Furthermore, while EZH2 progressively increases in advanced disease, ATF3 levels progressively decrease, consistent with the finding that ATF3 is a direct EZH2 target (Fig 4A, left panel reproduced from Fig 1A for comparison purposes, S6 Data). Indeed, EZH2 and ATF3 levels are significantly inversely correlated with each other, albeit modestly (S4 Fig, Pearson correlation = −0.19, S7 Data). However, this analysis does not take into account the activity of HDACs, which are heterogenesously overexpressed in prostate cancer, and can also influence the expression of PRC2 targets, including ATF3, as shown throughout.

Therefore, we examined the relationship between PRC2 transcriptional signatures and ATF3-trancriptional signatures in multiple prostate cancer mRNA datasets, to determine if there was an association between effective PRC2 derepression and ATF3 activity. Strikingly, we found that the expression of PRC2 target genes (e.g., more effective derepression) strongly and significantly correlated with high ATF3 transcriptional activity (Pearson correlation = 0.74, Fig 4C and S6 Data). This finding was consistent in 2 additional datasets (Fig 3D, Pearson correlation = 0.4217; Fig 3E, Pearson correlation = 0.3592, S6 Data). Finally, we assessed the expression of ATF3-regulated genes in tumors with low levels of EZH2 (bottom 10%) and tumors with high levels of EZH2 (top 10%), in these 3 prostate cancer datasets (Fig 4F–4H). Importantly, tumors with the highest levels of EZH2 consistently exhibited lower expression of ATF3-regulated genes (Fig 4F–4I). These data further support the notion that the stress response gene, *ATF3*, is suppressed by these two important epigenetic enzymes during prostate cancer progression. Moreover, its reactivation is a critical mediator of the therapeutic response to combined EZH2 and HDAC inhibitors, which represent a viable therapeutic strategy for castration-resistant disease.

## Discussion

The primary means of treating metastatic prostate cancer has been to target androgen signaling. Treatments have progressed from medical to chemical ablation of androgen signaling and currently include agents that directly target the AR (e.g., enzalutamide) [2,3]. Despite the initial effectiveness of these therapies, if diagnosed at an advanced stage, tumors inevitably become resistant to androgen deprivation strategies and are lethal. Therefore, we have been focusing on developing therapeutic strategies that target pathways other than the AR, in order to develop additional treatment options for individuals with CRPC.

Here, we describe a promising therapeutic strategy for CRPC that co-targets two major epigenetic enzymes in CRPCs: EZH2 and HDAC. Specifically, we show that agents that inhibit both enzymes are required to reprogram castration-resistant tumors and effectively derepress a subset of PRC2 targets. One of these targets, *ATF3*, is a common stress response gene that is up-regulated in response to numerous cellular and environmental insults, many of which occur during tumor development and progression [34]. Importantly, ATF3 up-regulation has been shown to be functionally required for cell death triggered by these insults and in response to chemotherapies [20–24]. Therefore, we speculate that the epigenetic suppression of *ATF3* might be essential for survival as tumors are exposed to increasing cellular and environmental stresses associated with tumor development, progression, and metastasis. This model is consistent with the observation that loss of *ATF3* enhances prostate cancer development and progression in GEMMs [32,33] and that ATF3 levels becomes progressively suppressed in advanced human tumors (shown herein).

It should be emphasized that EZH2 and HDAC inhibitors effectively kill CRPCs in vitro and trigger frank tumor regression in vivo rather than cytostasis, which is typically not observed in castration-resistant models. Moreover, these agents are effective in models of CRPC that express AR but are insensitive to androgens, as well as those that lack AR altogether, indicating that this response is not dependent on AR suppression. Accordingly, this strategy may be suitable for several different types of castration-resistant disease. Its potential broad utility is further underscored by the fact that more than 92% of CRCPs overexpress EZH2. While we were not able to directly assess the effects of these agents on metastatic lesions with the models used in this study, it should be noted that these agents were effective in several CRPC tumor models, which grew at ectopic sites.

This drug combination should also be highly translatable. HDAC inhibitors are FDA approved for many hematopoietic malignancies [35]. Several EZH2 inhibitors are currently being investigated in clinical trials and appear to be well tolerated, and one (tazemetostat) has been approved for epitheliod sarcomas [9,10]. However, this study also provides one mechanistic explanation for why EZH2 and HDAC inhibitors are not effective as single agents in prostate cancer. EZH2 has been shown to play a causal role in driving prostate cancer development and progression, by suppressing key PRC2 target genes, some of which are known and many others which are likely unknown [6,36,37]. HDAC genes have also been shown to be overexpressed in prostate cancer [13,15]. The findings presented here suggest that EZH2 and HDACs play a cooperative role in maintaining the suppression of PRC2 targets and that a subset can only be derepressed in the presence of both agents. ATF3, a stress response gene that triggers cell death, is one of those genes. Regardless, these studies demonstrate that, together, these agents function via a cooperative mechanism of transcriptional reprogramming and represent a promising therapeutic strategy for this currently untreatable disease. The unbiased identification of ATF3 in this study highlights the important role that this stress response gene plays in the death of prostate cancers.

## Materials and methods

### Lead contact and material sharing

Further information and requests for resources and reagents should be directed to and will be fulfilled by the Lead Contact, Karen Cichowski (kcichowski@rics.bwh.harvard.edu). This study did not generate new unique reagents.

### Manual cell counting

To measure cellular proliferation and cytotoxicity, manual cell counting expeirments were performed. On day −6, cells were seeded at 40% to 50% confluency in 10 cm dishes. On day −5, cells were treated with GSK126 or DMSO. On day −3, cells were passaged to be at 40% to 50% confluency and maintained in GSK126 or DMSO. On day −1, cells were seeded at 150,000 cells (PC3, C42B, LnCAP) or 15,000 cells (PT-09) in 6-well tissue culture dishes or at 250,000 cells in 6 cm dishes for protein or RNA harvest. On day 0, cells were either counted using a hemocytometer, or treated with DMSO, Vorinostat, GSK126, or the combination in media with 10% charcoal stripped fetal bovine serum (Gibco, Cat # 12676029). On day 1, cells were harvested for RNA or protein. On day 4, remaining cells were counted using a hemocytometer, and log_2_ fold change to day 0 counts were calculated.

### In vitro drug treatments

Drug concentrations were as follows except where otherwise specified: GSK-126 (5 μM, Selleckchem, *S7061*), vorinostat (2 μM, Selleckchem, #S1047), panobinostat (50 nM, Selleckchem, #S1030), MAK683 (5 μM, Selleckchem, S8983), docetaxel (10 nM, Selleckchem, S7787).

### Synergy analysis

To measure synergistic interactions between EZH2i GSK126 and HDACi vorinostat, PC3 cells were analyzed by cell titer glo. On day −6, cells were seeded at 40% to 50% confluency in 10 cm dishes. On day −5, cells were treated with increasing doses of GSK126 (0, 1.25, 2.5, 5, 10 μM). On day −3, cells were passaged to be at 40% to 50% confluency and maintained in the same dose of GSK126. On day −1, 10,000 cells were seeded into 96-well plates. On day 0, cells were treated with the same doses of GSK126 and additionally, increasing doses of vorinostat (0, 0.5, 1, 2, 4, 8 μM) in media containing 10% charcoal-stripped fetal bovine serum. After 96 hours, cell viability was measured using CellTiter-Glo (Promega, #G9291) and normalized to DMSO to determine the response. SynergyFinder was used to calculate the synergy score using Gaddum’s noninteraction model—HSA. A score above 10 indicates synergistic interaction.

### Incucyte live cell imaging

Live cell imaging technology was used to measure whether the combination induces apoptosis in PC3 cells. After 5 days of pretreatment with GSK126, cells were seeded into 96-well plates with 10,000 cells per well. On day 0, cells were treated with GSK126 and/or vorinostat in media containing 10% charcoal-stripped fetal bovine serum, NucLight Rapid Red Reagent (Sartorius, #4717) to identify nuclei, and NucView Caspase-3 Enzyme substrate (VWR, 71003–852) to identify apoptotic cells. Cells were placed inside the IncuCyte live cell imager, and images were acquired every 2 hours for 72 hours. Percent apoptotic cells were measured using the built-in imaging software and calculating the number of cells positive for NucLight Rapid Red and NucView Caspase-3 divided by number of cells positive for NucLight Rapid Red alone.

### Cell lines

Cell lines were purchased directly from ATCC. PT-09 cells were generously provided by the Ruffell Lab at Moffitt Cancer Center. Specifically, they were derived from a tumor that developed in *Trp53*^*lox*^, *Pten*^*loxP*^, Pbsn-cre/Esr1* mice upon tamoxifen treatment and were determined to be castration resistant. PT-09, 293T, and LHS-AR cells were cultured in DMEM (Corning, # 10-013-CV), and LNCaP, PC3, and C42B cells were cultured in RPMI (Corning, # 10-040-CV). All media was supplemented with 10% fetal bovine serum and 1X concentration of Pen/Strep/Glutamine (Gibco, # 10378016).

### Western blotting

Cell lysates were collected by boiling pellets in hot 1% SDS lysis buffer (1% SDS, 100 mM NaCl, 10 mM Tris (pH 7.5)) for 5 minutes. Proteins were resolved by SDS-PAGE gels and transferred to PVDF membranes before blocking with 5% milk in TBST for 1 hour. Membranes were incubated with primary antibody overnight at 4°C and appropriate HRP-conjugated secondary antibody after washing with TBST. HRP signal was detected using film. Antibodies were purchased from Cell Signaling Technologies for H3K27me3 (cat# 9733S, RRID AB_2616029), H3K27ac (cat# 4353S, RRID AB_10545273), H3 (cat# 4499S, RRID AB_10544537), GAPDH (cat# 2118S, RRID AB_ 561053), SUZ12 (cat# 3737S, RRID AB_2196850), ATF3 (cat# 18665S, RRID AB_2827506), pAMPKα (cat# 2535S, RRID AB_331250), and AMPKα (cat# 2532S, RRID AB_330331), or from BD BioSciences for EZH2 (#612666, RRID AB_2102429).

### Subcutaneous xenografts and allografts

Animal procedures were approved the Center for Animal and Comparative Medicine in Harvard Medical School in accordance with the NIH Guide for the Care and Use of Laboratory Animals and the Animal Welfare Act and was approved by an Instiutional Animal Care and Use Committee under protocol 2016N000467 to Karen Cichwoski. PC3 cell line xenograft experiments were performed in Nu/Nu mice purchased from Charles River Laboratories, and PT-09 cell line allograft experiments were performed in C57BL6/J mice purchased from Jackson Laboratories. Briefly, cells in 50% Matrigel/media (1,000,000 cells for PC3; 500,000 cells for PT-09) were injected subcutaneously in the left and right flank of each mouse. Pretreatment with GSK-126 for 1 week began when tumors reached approximately 150 mm^3^, approximately 2 to 4 weeks after injection. For all mouse experiments, vorinostat was administered at 50 mg/kg by intraperitoneal injection (vehicle: 10% 2-hydroxypropyl cyclodextrin) daily. GSK-126 was administered at 300 mg/kg by intraperitoneal injection (vehicle: 10% Captisol) 2× weekly. Compounds given in combination were administered 8 hours apart. Tumor size was measured every 2 to 3 days by calipers, and volume was calculated using the standard formula L × W^2^ × π/6. Under IACUC guidelines, euthanasia was carried out using carbon dioxide overdose followed by cervical dislocation.

### Immunohistochemistry

Tumors were harvested for IHC 4 hours after final dose of drug treatment by removing tumor and fixing in Formaldehyde-Fresh (Fischer Scientific, # SF94-4) for 24 hours. After 24 hours, tumors were stored in 70% ethanol before sectioning and analysis. Sectioning was performed at the Harvard Medical School Rodent Histopathology Core. For CC3 staining, antigen was retrieved in Tris-EDTA buffer (10 mM Tris Base, 1 mM EDTA, 0.05% Tween 20 (pH 9.0)) at 95°C for 10 minutes and then blocked for 1 hour in blocking solution (5% goat serum, 1 mg/mL BSA, 0.05% Tween 20, 1X PBS). Primary antibody against CC3 (CST 9664S) at a 1:500 dilution was incubated with the sections for 1.5 hours before washing, incubation with secondary antibody for 1.5 hours, and development with DAB for 3.5 minutes.

### Microarray RNA expression analysis

PC3 cells were treated with either vehicle or GSK-126 (5 μM) for 5 days, followed by vehicle or vorinostat (2 μM) for 24 hours. RNA was isolated using TRIzol, following the manufacturer’s protocol, and RNA cleanup was performed using the Qiagen RNeasy kit (#74104). The Molecular Biology Core Facilities at Dana-Farber Cancer Institute hybridized RNA to the Affymetrix Human 2.0 STS array chip. Raw CEL file results were extracted and normalized using the Affymetrix Transcription Analysis Console software. Genes significantly induced by the combination were defined as log_2_ fold change > 1 and Benjamini–Hochberg corrected *p*-value < 0.05. To find cooperatively induced genes induced by the combination, we found genes that were significantly induced between combo and each of the single treatment or control treatment arms. Differentially induced genes were defined as log_2_ fold change > 1 and Benjamini–Hochberg corrected *p*-value < 0.05 in all 3 conditions of combo versus DMSO, combo versus HDACi, and combo versus EZH2i.

### Gene set enrichment analysis

Single sample GSEA was performed using GenePattern (https://www.genepattern.org/). GSEA was performed using software available at http://www.gsea-msigdb.org/. ATF3 gene signature was ATF3_Q6 and EZH2 gene signature was NUYTTEN_EZH2_TARGETS_UP [38]. For ssGSEA, scores were presented as z scores.

### siRNA

For siRNA experiments, cells were transfected with 20 μM siRNA using a 1:400 dilution of Lipofectamine RNAiMAX (Invitrogen, cat # 13778–075). Transfection occurred on day −1 for manual counting experiments. After 6 hours of incubation, cells were seeded as described above. siRNA was purchased from Horizon Biosciences for PLA2G2A (L-009901-00), GPR160 (L-005520-00), LIMCH1 (L-024200-00), GABRG1 (L-006173-00), STC1 (L-006477-00), SCGB2A1 (L-019606-01), NCKAP1L (L-019219-01), EGLN3 (L-004274-00), TNFSF9 (L-011525-00), SLITRK4 (L-017734-01), ATF3 (D-008663-00-0005), or a nontargeting control (D-001810-10-50).

### qPCR

Quantitative PCR was used to measure gene expression in response to the combination. RNA was isolated from cells after 24 hours of combination treatment (day 1) using RNeasy Plus Kit (Qiagen, Cat # 74134). RNA was reverse transcribed into cDNA using the High-Capacity cDNA Reverse Transcription kit (Thermo Fischer Scientific, cat # 4368814). qPCR was completed using the PerfeCTa SYBR Green SuperMix Reaction Mix (QuantaBio, cat # 95054–500). Cq values were normalized to STAU1 for human samples and GAPDH for mouse samples. Primers are reported in S2 Table.

### ChIP-seq

ChIP-seq was performed on cells treated with either vehicle or GSK-126 (5 μM) for 5 days, followed by vehicle or vorinostat (2 μM) for 24 hours. Antibodies used were H3K27ac (Abcam, Cat. #ab4729) and H3K27me3 (Milipore #07–449). Cells were crosslinked in 1% formaldehyde at RT for 10 minutes while gently shaking and then quenched in 0.125M Glycine for 5 minutes at RT. Cells were then washed twice in ice cold 1× PBS. Protease inhibitor cocktail (PIC) was added to all the lysis and wash buffers. Pelleted cells were resuspended and lysed while rocking at 4°C for 10 minutes in 3 ml (per 10^7^ to 30^7^ cells) of lysis buffer 1 (LB1), containing 50 mM Hepes-KOH (pH 7.5), 140 mM NaCl, 1 mM EDTA, 10% Glycerol, 0.5% Igepal, 0.25% Troton X-100. The lysates were spun at 1,350 × *g* for 5 minutes at 4°C and supernatant discarded. Pellets were resuspended in 3 ml of lysis buffer 2 (LB2), containing 10 mM Tris–HCL (pH 8.0), 200 mM NaCl, 1 mM EDTA, and 0.5 mM EGTA. Lysates were incubated in LB2 for 10 minutes at RT and then spun at 1,350 × *g* at 4°C for 5 minutes. Supernatant was discarded. Finally, pellets were resuspended in lysis buffer 3 (LB3), containing 10 mM Tris–HCl (pH 8.0), 100 mM NaCl, 1 mM EDTA, 0.5 mM EGTA, 0.1% Na-Deoxycholate, and 0.5% N-lauroylsarcosine. Cells were transferred to polypropylene tubes and sonicated to yield fragments sized 200 to 500 bp. One percent each sample was frozen on dry ice to be saved as the input control. Lysates were incubated with H3K27-3me Antibody overnight in the presence of the precleared magnetic beads. Next day, the beads/Ab/lysates mixes were washed 3 times in low salt buffer, containing 0.1% SDS, 1% Triton X-100, 2 mM EDTA, 20 mM Tris–HCl (pH 8.1), 150 mM NaCl, then 3 times in high salt buffer, containing 0.1% SDS, 1% Triton X-100, 2 mM EDTA, 20 mM Tris–HCl (pH 8.1), 500 mM NaCl, and then 3 times in LiCl buffer, containing 0.25M LiCl, 1% NP-40, 1% Deoxycholate, 1 mM EDTA, 10 mM Tris–HCl (pH 8.1), and finally, once in ice-cold TE buffer. Beads were eluted and cross-links reversed overnight at 65°C in elution buffer, containing 50 mM Tris–HCl (pH 8.0), 10 mM EDTA (pH 8.0) and 1% SDS. Input pellets were thawed out, and cross-linking was similarly reverse overnight in elution buffer. Next day, RNA and cellular protein were digested with RNase A (0.2 mg/ml) at 37°C for 2 hours and proteinase K (0.2 mg/ml) at 55°C for 30 minutes. DNA was isolated with phenol:chloroform:isoamyl alcohol, and concentration was measured with Nanodrop. Isolated DNA was submitted to the Center for Cancer Computational Biology (CCCB) at Dana Farber Cancer Institute for the library preparation and sequencing.

### ChIP-seq read mapping and peak calling

Reads were initially trimmed using cutadapt [39] in single-end mode with the parameters -e 0.1 -q 16 -O 3 –trim-n–minimum-length 25 -a AGATCGGAAGAGC. Trimmed reads were then aligned to the hg19 reference genome using the bwa-mem algorithm [40] with default parameters. Aligned ChIP-seq reads were then analyzed using HOMER [41] as follows: (1) tag directories were made using the makeTagDirectories command with default parameters and the sorted bam file for each sample as input; and (2) peaks were found using the findPeaks command with the following parameters: -style histone -minDist 2000 and using the input DNA for each drug treatment as the control tag directory. Bedgraph files for visualization in UCSC Genome Browser [42] were made using the HOMER command makeUCSCfile -fsize 50e6 for each tag directory.

### ChIP-seq differential peak analysis

We considered HOMER-called peaks that were within a 20-kb window (+/− 10 kb) of a protein-coding transcript start site (as annotated in Gencode v19; [43]); to do this intersection, we used the bedtools [44] intersect command with default parameters. Transcripts were considered to have peaks “lost” in a given condition if at least 1 peak within the 20-kb window were lost compared to the differential condition control (usually DMSO-treated samples unless otherwise noted). A gene was considered to have peaks “lost” in a given condition if at least 1 transcript met the aforementioned requirements. List of 2,258 genes identified as genes who lost H3K27me3 in response to EZH2i (PRC2 target genes) is reported in S1 Table.

### Reactive oxygen species assays

ROS levels were detected using the ROSglo Assay Kit (Promega, cat # G8820) after 24 hours of combination treatment. ROS levels were normalized to DMSO-treated controls.

### ATP measurements

ATP levels were detected using the CellTiter-Glo Luminescent Assay (Promega, cat # G7570) after 8 hours of combination treatment (before onset of cell death). ATP levels were normalized to DMSO-treated controls.

### ATF3 sgRNA mediated knockdown

ATF3 was knocked down using CRISPR-mediated gene editing. Cells were transduced using pLentiCRISPR-V2 (Addgene 52961) and selected with puromycin (1 μg/mL) for 2 days. sgRNA sequence for ATF3 was GGAGCCCGGACAATACACGT.

### Publicly available datasets

Relative expression of EZH2 and ATF3 (Figs 1A and 4A–4C) and survival data were obtained from Grasso and colleagues (doi: 10.1038/nature11125) [45]. EZH2 and ATF3 expression and related transcriptional signatures were analyzed using data from Taylor and colleagues (doi: 10.1016/j.ccr.2010.05.026) [46], TCGA Firehose (doi: 10.7908/C1MK6CC8) [46], and Abida and colleagues (doi: 10.1073/pnas.1902651116) [47]. EZH2 high and low tumors were categorized as the top 10% and bottom 10% of all tumors within the dataset.

## Supporting information

S1 Fig Related to Fig 1. **(A)** Graph depicting IC_50_ scores of vorinostat (HDACi) in PC3 cells treated with DMSO or EZH2i (S5 Data). (**B, C**) Bar graphs depicting effects of DMSO, GSK-126 (EZH2i), vorinostat (HDACi), or both agents on the number of immortalized prostate epithelial cells (**B**) and the non-prostatic cancer HEK293T cell line (**C**) over time as determined by manual counting. The y-axis indicates the log_2_ fold change in cell number after 4 days of treatment relative to day 0. Data are mean ± SD of biologically independent samples (S5 Data). (**D**) Immunoblot depicting pharmacodynamic study of xenograft tumors in response to specified treatments shown in Fig 1G and 1H. Effects on H3K27ac and H3K27me3 are shown. GAPDH serves as loading control. (**E**) Tumor weight of PT-09 tumors after 28 days of treatment (S5 Data).
(TIFF)

S2 Fig Related to Fig 2. **(A**) Metaplot of H3K27me3 ChIP-seq data from PC3 cells treated with DMSO, EZH2i, HDACi, or combo. Transcription start sites are centered in each plot. (**B**) Heatmap of 68 genes differentially transcriptionally induced by the combination (significantly different genes in all 3 comparisons: combo vs. DMSO, combo vs. HDACi, combo vs. EZH2i). Genes labeled in blue are 21 genes whose promoters lost H3K27me3 in response to EZH2i. (**C**) Table describing rationale for how 11 genes (out of 21 candidate genes) were chosen for inclusion in siRNA screen.
(TIFF)

S3 Fig Related to Fig 3. **(A)** qPCR for ATF3 mRNA expression in 3 prostate cancer cell lines treated with DMSO, EZH2i, HDACi, or the combination. ATF3 mRNA levels were first normalized to housekeeping gene STAU1, and then fold change was calculated relative to DMSO. Data are mean ± SD of biological independent samples. *P* values measured by unpaired one-tailed homoscedastic Student *t* test (S6 Data). (**B**) Heatmap of differentially expressed genes within ATF3_Q6 gene set. (**C**) Western blot associated with manual cell counting data from Fig 3C. C42B cells were transfected with siRNA against ATF3 or a control sequence. Cells were treated with DMSO, 5 μM GSK126 (EZH2i), 2 μM vorinostat (HDACi), or a combination of the 2 inhibitors. (**D**) Quantification of ATP levels after 8 hours of treatment with DMSO, EZH2i, HDACi, or the combination using CellTiter-Glo. Box plot reports the mean of 5 biologically independent samples with whiskers reporting minimum and maximum. *P* values measured by unpaired one-tailed homoscedastic Student *t* test (S6 Data).
(TIFF)

S4 Fig Related to Fig 4. Correlation plot of EZH2 mRNA and ATF3 mRNA expression in Taylor and colleagues dataset [46] (S7 Data).
(TIFF)

S1 Table List of 2,258 genes identified as genes who lost H3K27me3 in response to EZH2i (PRC2 target genes). (XLSX)

S2 Table Table of primer sequences used for RT-qPCR. (XLSX)

S1 Data Numerical data for Fig 1A, 1B, 1D, 1E, 1F, 1G, 1H and 1J. (XLSX)

S2 Data Numerical data for S1A, S1B, S1C and S1E Fig. (XLSX)

S3 Data Numerical data for Fig 2A, 2B, 2C, 2D and 2G. (XLSX)

S4 Data Numerical data for Fig 3A, 3C, 3D, 3F, 3I, 3J and 3L. (XLSX)

S5 Data Numerical data for S3A and S3D Fig. (XLSX)

S6 Data Numerical data for Fig 4A, 4B, 4C, 4D and 4E. (XLSX)

S7 Data Numerical data for S4 Fig. (XLSX)

S1 Raw Images Images of complete blots for all immunoblots. (PDF)

## Abbreviations

AR: androgen receptor
CC3: cleaved caspase 3
ChIP-seq: chromatin immunoprecipitation-sequencing
CRPC: castration-resistant prostate cancer
EZH2: enhancer of zeste homologue 2
GSEA: gene set enrichment analysis
HDAC: histone deacetylase
PRC2: Polycomb Repressive Complex 2
ROS: reactive oxygen species
RT-qPCR: quantitative reverse transcription PCR
