# Supplementary material for: Combating castration-resistant prostate cancer by co-targeting the epigenetic regulators EZH2 and HDAC
Source: PLoS Biol. 2023 Apr 27;21(4):e3002038. doi: 10.1371/journal.pbio.3002038 (PMC10138213; doi:10.1371/journal.pbio.3002038)

Raw immunoblots

Note: same samples were run on separate gels

Related to Fig. 1B

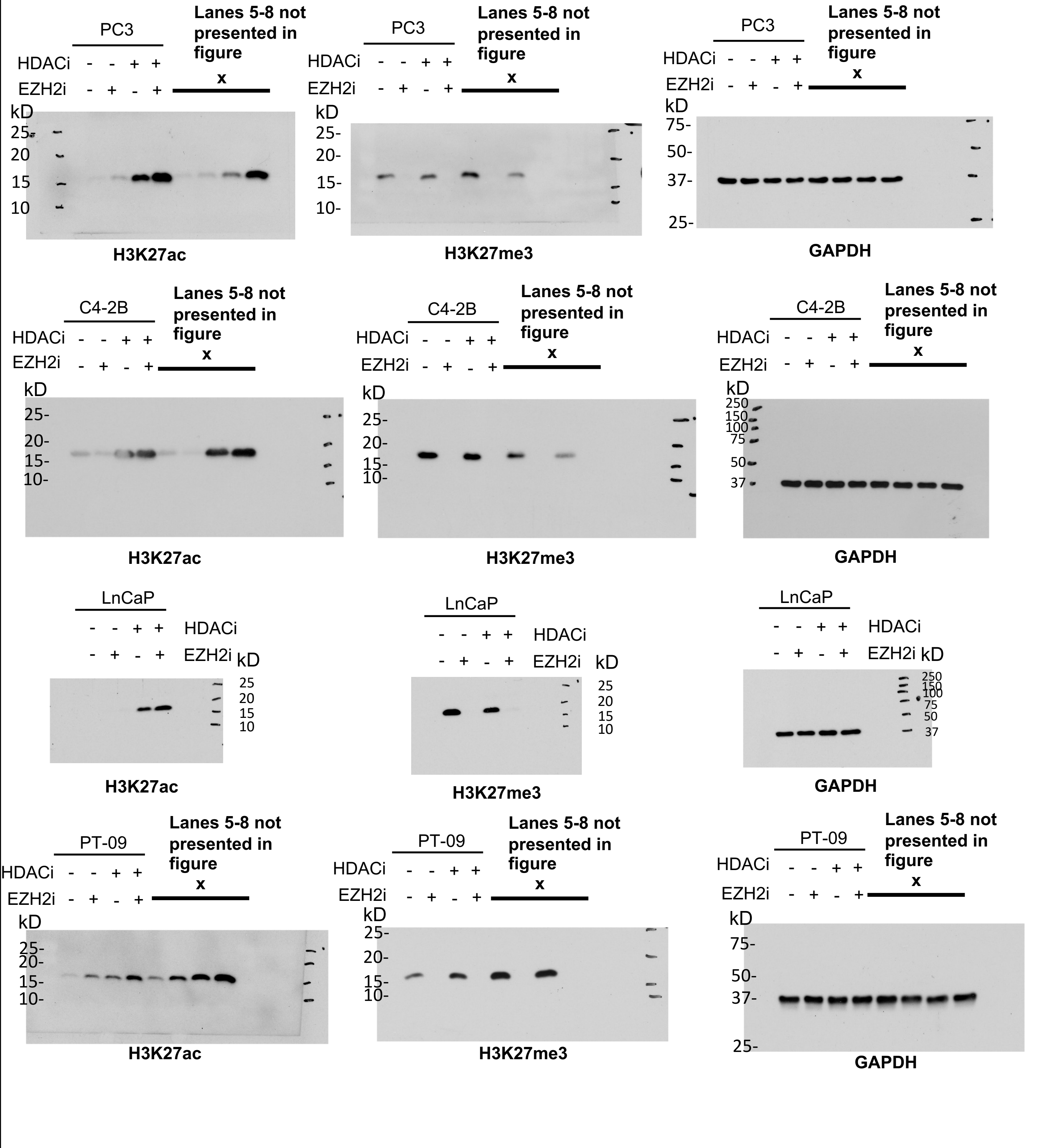

Related to Fig. 1F

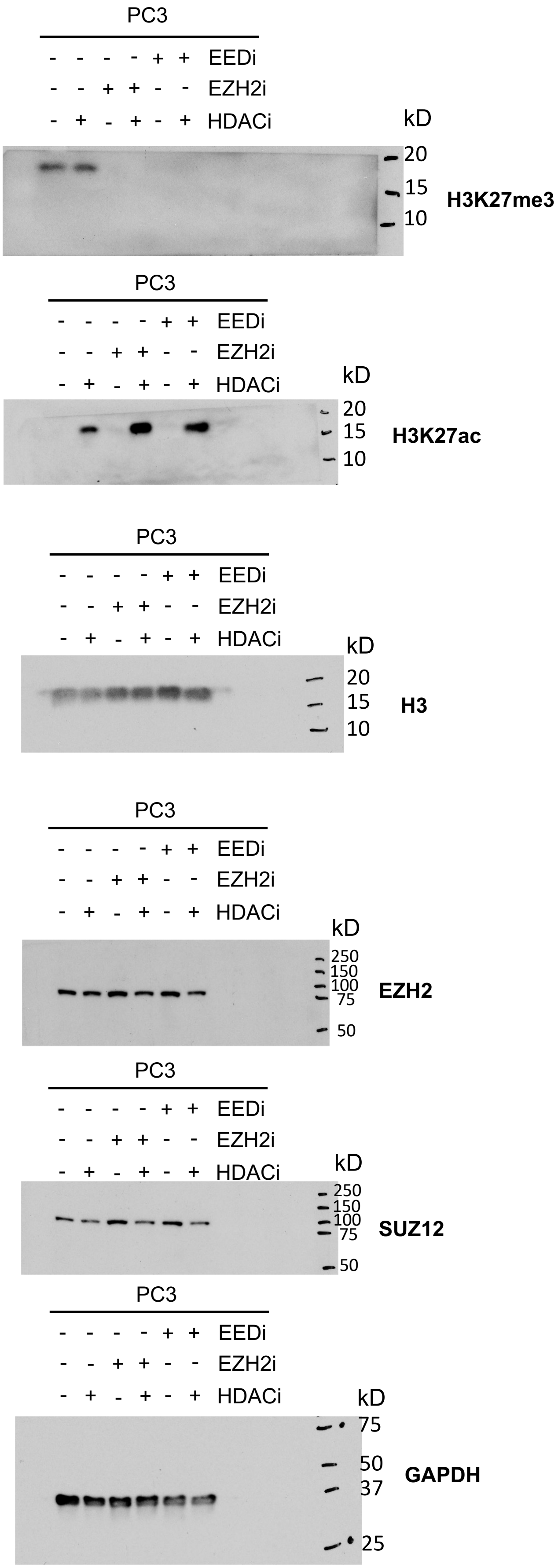

Related to Supplemental Fig. 1D

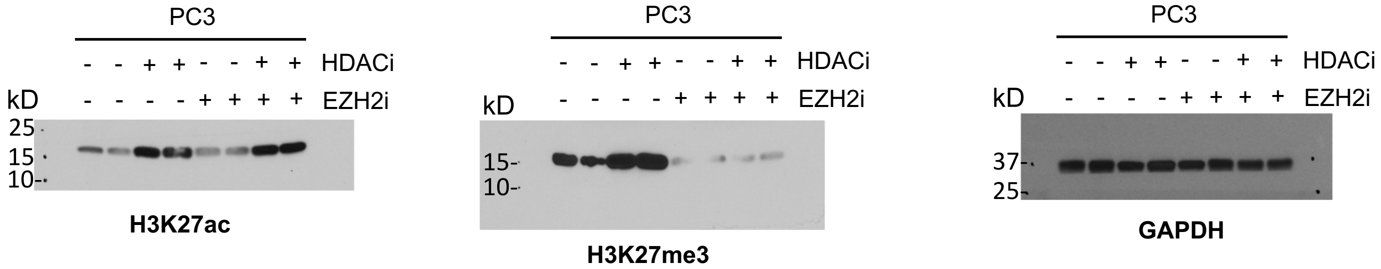

Related to Fig. 3A

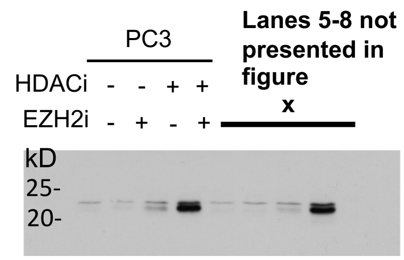

ATF3

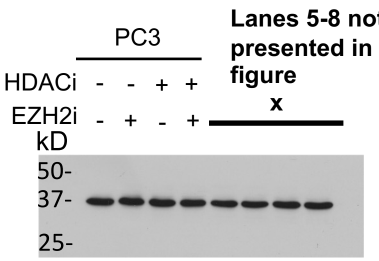

GAPDH

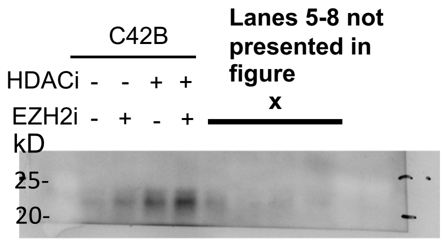

ATF3

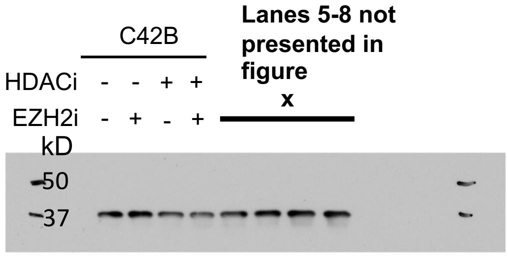

GAPDH

Related to Fig. 3E

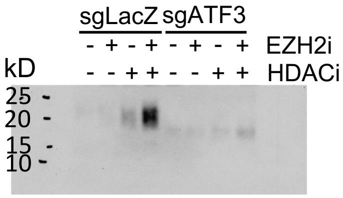

ATF3

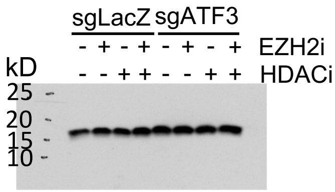

H3

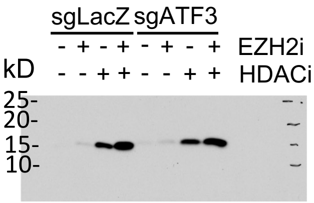

H3K27ac

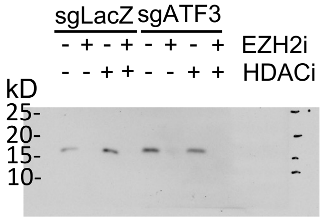

H3K27me3

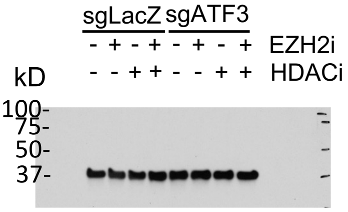

GAPDH

Related to Fig. 3H

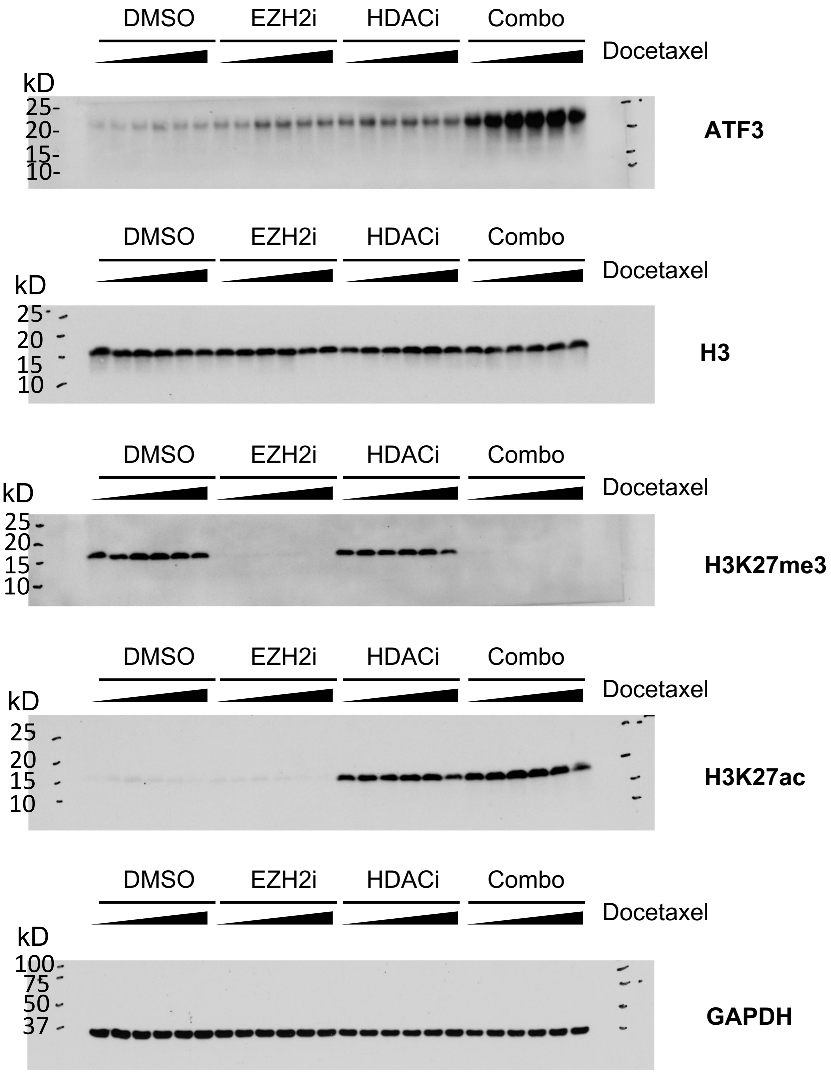

Related to Fig. 3I

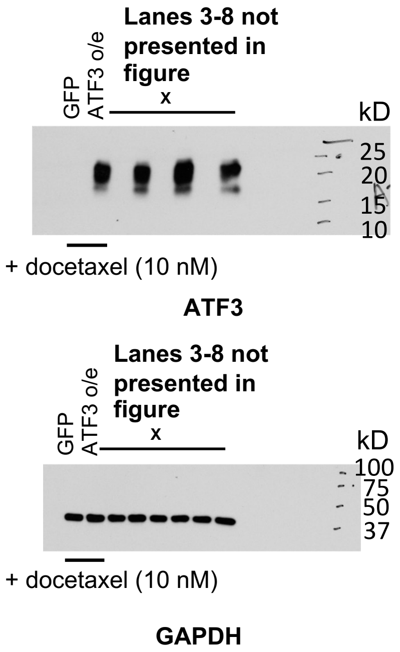

Related to Fig. 3K

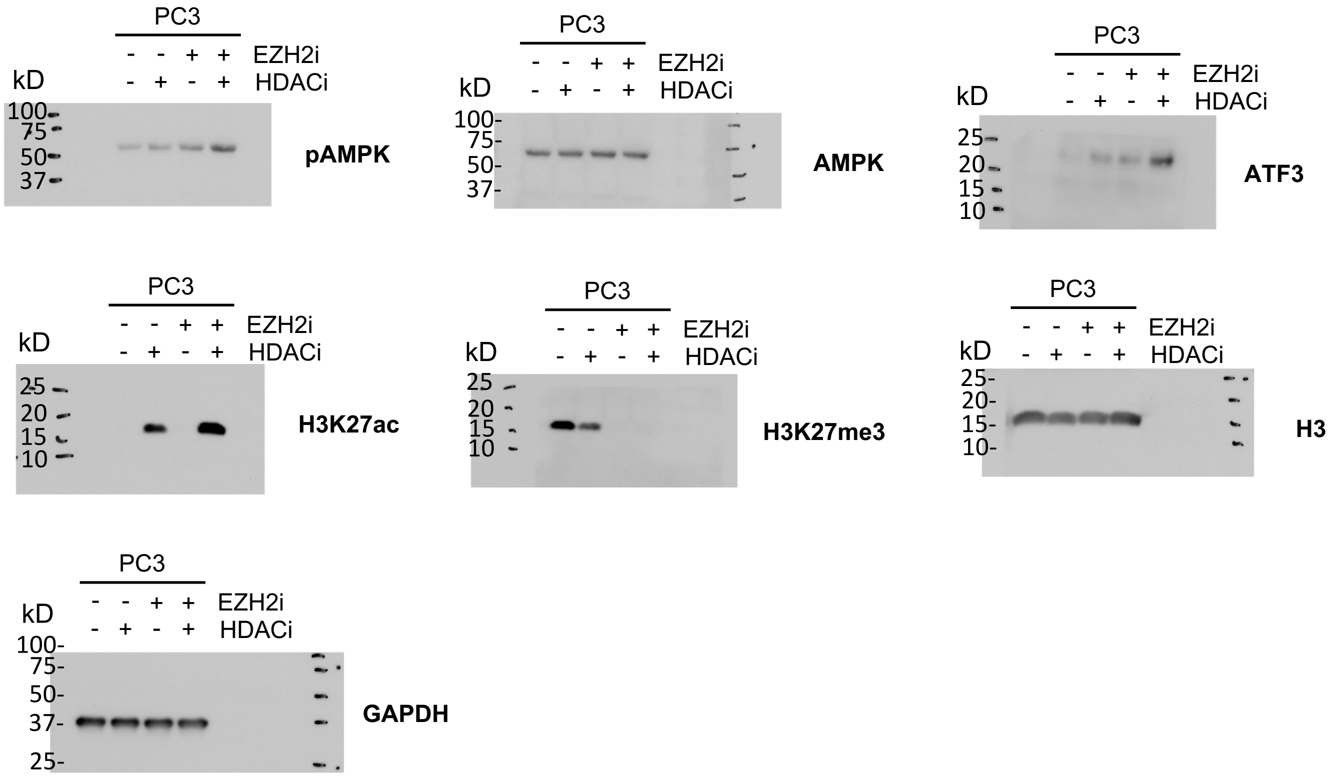

Related to Fig. 3L

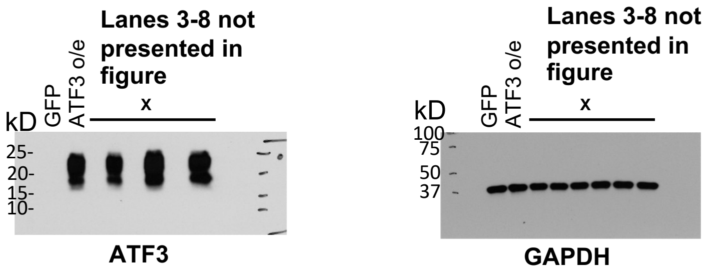

Related to Supplemental Fig. 3 C42B siATF3

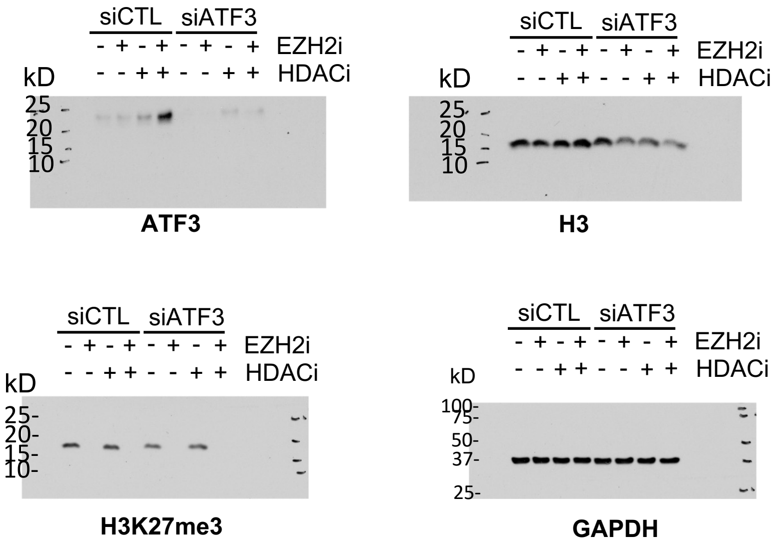

Supplement: S1 Raw Images — (PDF) [file pbio.3002038.s014.pdf]
